# Defining a critical therapeutic window: cyclosporine exposure in months 1–2 as a determinant of acute graft-versus-host disease in patients with acute leukemia undergoing allogeneic hematopoietic stem cell transplantation from matched related donors

**DOI:** 10.1007/s00277-026-07131-9

**Published:** 2026-06-15

**Authors:** Neslihan Hazel Önür, Vildan Özkocaman, Fahir Özkalemkaş

**Affiliations:** 1 Department of Internal Medicine, Bursa Uludag University Faculty of Medicine, Bursa, Türkiye; 2 Division of Hematology, Department of Internal Medicine, Bursa Uludag University Faculty of Medicine, Bursa, Türkiye; 3 Division of Geriatrics, Department of Internal Medicine, Istanbul University Fatih, Istanbul, Türkiye

**Keywords:** Cyclosporine, Acute leukemia, Acute graft-versus-host disease, Allogeneic hematopoietic stem cell transplantation

## Abstract

Acute graft-versus-host disease (aGVHD) is one of the most fatal yet preventable complications of hematopoietic stem cell transplantation (HSCT). Identifying optimal cyclosporine (CsA) blood level thresholds is particularly important in patients receiving CsA-based prophylaxis; however, cut-off values remain unclear. This retrospective study evaluated 120 patients with acute leukemia (64% acute myeloid leukemia [AML], 36% acute lymphoblastic leukemia [ALL]) who underwent allogeneic HSCT (AHSCT) between June 2011 and March 2021. CsA combined with methotrexate was used for GVHD prophylaxis. Statistical analyses included ROC analysis to determine optimal cut-off values and logistic regression to identify independent risk factors for aGVHD. aGVHD developed in 34 patients (28%), with a median onset at day 48. ROC curve analysis identified a CsA cut-off level of 274 µg/L during months 1–2, with an AUC of 0.697 (95% Confidence Interval [CI]: 0.574–0.820, *p* = 0.005), sensitivity of 77.3%, and specificity of 63%. In multivariate analysis, higher CsA levels during months 1–2 (Odds Ratio [OR] = 0.990, 95% CI 0.981–1.000; *p* = 0.039) and pre-transplant steroid use (OR = 0.284, 95% CI 0.094–0.860; *p* = 0.026) were independently associated with reduced risk of aGVHD. No universally accepted cut-off value of CsA levels has been defined to date, and our findings suggest that maintaining levels above 274 µg/L during the first 1–2 months post-transplant is critical to reduce aGVHD risk.

## Introduction

Allogeneic hematopoietic stem cell transplantation (AHSCT) has become a potentially curative treatment option for a wide range of hematologic and non-hematologic disorders, including both malignant and non-malignant diseases [1–3]. Despite its curative potential, the clinical utility of AHSCT is constrained by life-threatening complications that may occur in the post-transplant period [4]. Among these, acute graft-versus-host disease (aGVHD) represents one of the most feared and challenging complications, accounting for a major cause of transplant-related morbidity and mortality. aGVHD develops as a result of donor-derived T lymphocytes recognizing host alloantigens, which triggers immune-mediated injury in target organs, most commonly the skin, gastrointestinal tract, and liver [5, 6]. aGVHD typically occurs within the first 100 days after AHSCT and has been reported to affect approximately 30–50% of transplant recipients in previous studies [7].

Over the years, various preventive approaches have been introduced in both the pre- and post-transplant settings to mitigate this risk and to improve overall transplant success. These protocols mainly involve immunosuppressive regimens aimed at suppressing T-cell activation and proliferation, thereby reducing the incidence and severity of aGVHD [3]. Cyclosporine (CsA), a calcineurin inhibitor that prevents T-cell activation by blocking interleukin-2 transcription, has long been considered a cornerstone agent in GVHD prophylaxis [3]. In clinical practice, CsA is frequently administered in combination with methotrexate (MTX), and this regimen remains a commonly used approach for GVHD prevention in allogeneic transplantation [5, 8, 9].

However, the clinical use of CsA is often challenging due to its narrow therapeutic window and significant risk of toxicity. Previous studies have demonstrated that low CsA levels are associated with an increased risk of aGVHD, whereas excessively high levels may lead to adverse effects such as nephrotoxicity, neurotoxicity, and hypertension [4, 10–14]. Therefore, determining the optimal CsA levels is of critical importance for post-transplant outcomes, as they directly influence the risk of complications and long-term survival.

Given these considerations, the present study was designed to investigate the association between early post-transplant CsA blood levels and the subsequent development of aGVHD in patients undergoing AHSCT for acute leukemia. Specifically, we analyzed CsA concentrations across four predefined time intervals within the first 100 days (0–14 days, 1st month, 1–2 months, and 2–3 months) in order to identify threshold levels predictive of aGVHD. By determining clinically relevant cut-off values, our study aims to provide practical guidance for optimizing immunosuppressive management and improving post-transplant outcomes.

## Methods

### Study population

Patients with acute leukemia who underwent AHSCT (*n* = 120; 64% acute myeloid leukemia [AML], 36% acute lymphoblastic leukemia [ALL]) between June 2011 and March 2021 at the Transplantation Unit of the Hematology Department, Bursa Uludağ University Faculty of Medicine Hospital, were retrospectively analyzed.

Inclusion criteria were being ≥ 18 years of age, having undergone AHSCT for acute leukemia, and having both the transplantation procedure and subsequent follow-up performed at the Hematology Department of Bursa Uludağ University Faculty of Medicine, with a minimum follow-up duration of 100 days after transplantation. Exclusion criteria were being < 18 years of age, undergoing AHSCT for non-malignant hematological disorders, undergoing autologous stem cell transplantation, or having the transplantation procedure and/or follow-up performed outside the Hematology Department of Bursa Uludağ University Faculty of Medicine.

### Patient characteristics

Collected baseline characteristics included age at transplantation, sex, height, weight, body mass index (BMI), diagnosis, disease risk category, treatment type, whether total body irradiation (TBI) or cranial radiotherapy (RT) was administered, conditioning regimen, donor-recipient HLA compatibility, blood type matching, GVHD prophylaxis and modifications, pre-transplant steroid use and cytomegalovirus (CMV) status after AHSCT.

### GVHD prophylaxis

GVHD prophylaxis consisted of a CsA and methotrexate (MTX) in all patients.

CsA administration: Intravenous CsA was initiated one day before transplantation at a dose of 3 mg/kg/day in two divided doses. Blood CsA levels were measured daily at 06:00, with target levels of 200–400 ng/mL. Dose titration (up to 3–5 mg/kg/day) was performed when target levels were not achieved. After mucositis resolved and oral intake was tolerated, CsA was switched to oral form, given twice daily according to blood levels. Blood CsA levels were monitored twice weekly during the first month of hospitalization. Patients were also closely monitored for clinical and laboratory findings suggestive of CsA toxicity.

MTX administration: Intravenous MTX was administered at 10 mg/m² on day + 1, and 5 mg/m² on days + 3 and + 6. Due to severe mucositis, day + 11 administration was omitted. Folinic acid rescue (30 mg IV, followed by two doses of 15 mg at 6-hour intervals) was given 24 h after each MTX dose.

In patients who failed to reach target CsA levels, developed high-grade toxicity, or did not improve despite dose adjustment or were unable to tolerate CsA prophylaxis was modified to mycophenolate mofetil (MMF) or, less commonly, tacrolimus (TAC). Some patients were re-initiated on CsA after toxicity resolution, while others continued alternative prophylaxis. Patients requiring permanent switch were excluded from analysis.

### Data collection and definitions

Data were obtained from outpatient visit records, inpatient charts, and the hospital automation system. Vital signs, physical examination findings, and laboratory results were reviewed. This study was conducted in accordance with the principles of the Declaration of Helsinki. Institutional Ethics Committee approval was obtained before data collection (Number: 2022-4/49). Written informed consent was obtained from all patients prior to transplantation and data usage for research purposes.

The diagnosis of aGVHD was based on clinical, laboratory, and pathology findings, and differential diagnoses were excluded.

Blood CsA levels were determined by chemiluminescent microparticle immunoassay (CMIA) using the Abbott Architect i2000SR analyzer (Abbott Diagnostics, USA). Blood CsA levels were averaged for four post-transplantation intervals: 0–14 days, 1st month, 1–2 months, and 2–3 months.

### Statistical analysis

Descriptive statistics included mean, standard deviation, median, minimum, maximum, frequencies, and percentages. Normality of continuous variables was assessed using Kolmogorov-Smirnov and Shapiro-Wilk tests. Comparisons between patients with and without aGVHD were performed using Pearson’s chi-square test, Fisher’s exact test, or Fisher-Freeman-Halton exact test for categorical variables, as appropriate. Monte Carlo simulation was applied when test assumptions were not met. Continuous variables with normal distribution were analyzed using the independent samples t-test, while non-normally distributed variables were analyzed using the Mann-Whitney U test.

Cut-off values of CsA predicting aGVHD were determined with ROC analysis using the Youden index. Since ROC analyses were performed for four different post-transplant time intervals, Bonferroni correction was applied for multiple comparisons, and adjusted p-values < 0.0125 were considered statistically significant. For ROC analyses, all patients were included. CsA levels were analyzed as fixed exposure variables within predefined post-transplant time windows, irrespective of the timing of aGVHD onset. Univariate and multivariate logistic regression analyses were performed to identify risk factors for aGVHD, with results reported as odds ratios (OR) and 95% confidence intervals (CI). Model fit was evaluated using the Hosmer-Lemeshow test.

All analyses were performed using SPSS version 26.0, and *p* < 0.05 was considered statistically significant.

### Use of artificial intelligence tools

During the preparation of this manuscript, ChatGPT (OpenAI, San Francisco, CA, USA) was used exclusively for grammar checking, language refinement, and formatting assistance. No AI tools were used for data generation, analysis, or interpretation. The authors reviewed and edited all AI-assisted content and take full responsibility for the final version of the manuscript.

## Results

A total of 120 patients with hematologic malignancies were included in the analysis, of whom 46 (38.3%) were female and 74 (61.7%) were male. The age of the patients at the time of transplantation ranged from 19 to 72 years, with a mean age of 41.3 ± 11.4 years and a median age of 42 years (Table 1).

The primary hematologic diagnoses of the patients were as follows: AML, ALL, AML transformed from MDS, and ALL transformed from CML, with 64 (53.3%), 38 (31.7%), 13 (10.8%), and 5 (4.2%) respectively. The disease risk categories were high risk, intermediate risk, low risk, and standard risk, with 54 (45%), 14 (11.7%), 1 (0.8%), and 51 (42.5%) patients, respectively. Prior to transplantation, 91 (75.8%) patients received standard therapy, while 29 (24.2%) patients received a combination of standard and salvage therapy. Allogeneic stem cell transplantations were performed using sibling donors only, with no unrelated donor transplantations included in the study cohort. The majority of patients had fully HLA-matched (10/10) sibling donors (*n* = 115, 95.8%), while a small proportion had HLA-mismatched (9/10) sibling donors (*n* = 5, 4.2%). The patients’ other comorbid conditions, transplant regimen, whether they received TBI and RT, HLA compatibility, stem cell source, and chimerism status are presented in Table 1.

aGVHD developed in 34 (28.3%) of the patients who underwent AHSCT. Among these patients, 11 (32.6%) were female and 23 (67.6%) were male (Table 1). Of the patients with aGVHD, 18 (52.9%) had grade I–II disease and 13 (38.2%) had grade III–IV disease, while grading information was unavailable for 3 (8.8%) patients. Skin was the most frequently involved organ (*n* = 13, 38.2%), followed by gastrointestinal involvement (*n* = 7, 20.6%) and liver involvement (*n* = 4, 11.8%). Multi-organ involvement was observed in 10 patients. Loss of chimerism was significantly more frequent in patients without aGVHD (15.1% vs. 0%, *p* = 0.010), whereas CMV positivity was more common among those who developed aGVHD (67.9% vs. 39.5%, *p* = 0.008). Moreover, pre-transplant steroid use was observed more frequently in patients without aGVHD (8.1% vs. 2.9%, *p* = 0.001) (Table 1). The incidence of aGVHD was significantly higher among patients who were switched from CsA to an alternative prophylactic agent due to intolerance or toxicity than among those who continued CsA throughout the prophylaxis period (*p* < 0.001).

**Table 1:** Baseline demographics and transplant-related characteristics of patients

Variables	aGVHD (-)	aGVHD (+)	Total	*p* value
	**86 (61.7%)**	**34 (28.3%)**	**120 (100%)**	
Sex (n,%)				0.532
Female	35 (40.7%)	11 (32.4%)	46 (38.3%)	
Male	51 (59.3%)	23 (67.6%)	74 (61.7%)	
Age (years) (mean±SD)	41±12	41±10	41±11	0.911
Weight (kg) (mean±SD)	75±15	77±13	75±15	0.380
Height (cm) (mean±SD)	168±9	169±9	168±9	0.297
BMI (kg/m2) (mean±SD)	27±5	27±5	27±5	0.714
Comorbidity (n,%)				
HT	6 (7%)	0 (0%)	6 (5%)	0.182
DM	3 (3.5%)	1 (2.9%)	4 (3.3%)	0.621
CRD	3 (3.5%)	2 (5.9%)	5 (4.2%)	1.000
Diagnosis (n,%)				0.139
AML	50 (58.1%)	14 (41.2%)	64 (53.3%)	
ALL	24 (27.9%)	14 (41.2%)	38 (31.7%)	
MDS > AML	10 (11.6%)	3 (8.8%)	13 (10.8%)	
KML > ALL	2 (2.3%)	3 (8.8%)	5 (4.2%)	
Risk Categories (n,%)				0.396
Low Risk	0 (0%)	1 (2.9%)	1 (0.8%)	
Intermediate Risk	11 (12.8%)	3 (8.8%)	14 (11.7%)	
High Risk	37 (43%)	17 (50%)	54 (45%)	
Standard Risk	38 (44.2%)	13 (38.2%)	51 (42.5%)	
Type of therapy prior to transplantation (n,%)				0.479
Standard	67 (77.9%)	24 (70.6%)	91 (75.8%)	
Standard + Salvage	19 (22.1%)	10 (29.4%)	29 (24.2%)	
Conditioning Regimen (n,%)				0.219
Bu-Cy	75 (87.2%)	26 (76.5%)	101 (84.2%)	
Cy-TBI	8 (9.3%)	7 (20.6%)	15 (12.5%)	
Flu-Cy-ATG	1 (1.2%)	0 (0%)	1 (0.8%)	
Flamsa	1 (1.2%)	0 (0%)	1 (0.8%)	
Vp-Cy-TBI	0 (0%)	1 (2.9%)	1 (0.8%)	
BEAM	1 (1.2%)	0 (0%)	1 (0.8%)	
TBI (n,%)	2 (2.3%)	1 (2.9%)	3 (2.5%)	1.000
Cranial RT (n,%)	4 (4.7%)	3 (8.8%)	7 (5.8%)	0.403
HLA Matching (n,%)				0.137
10/10	84 (97.7%)	31 (91.2%)	115 (95.8%)	
9/10	2 (2.3%)	3 (8.8%)	5 (4.2%)	
Stem Cell Source (n,%)				1.000
Peripheral Blood	85 (98.8%)	34 (100%)	119 (99.2%)	
Bone Marrow	1 (1.2%)	0 (0%)	1 (0.8%)	
Chimerism (n,%)				0.672
Present	79 (91.6%)	33 (97.1%)	112 (93.3%)	
Absent	6 (7%)	1 (2.9%)	7 (5.8%)	
Missing	1 (1.2%)	0 (0%)	1 (0.8%)	
Loss of Chimerism (n,%)				0.010*
Present	13 (15.1%)	0 (0%)	13 (10.8%)	
Absent	66 (76.7%)	33 (97.1%)	99 (82.5%)	
Missing	7 (8.1%)	1 (2.9%)	8 (6.7%)	
CMV Positivity (n,%)	34 (39.5%)	23 (67.9%)	57 (47.5%)	0.008*
Steroid Use Before AHSCT (n,%)	7 (8.1%)	1 (2.9%)	8 (6.6%)	0.001*
Prophylactic Agent (n,%)				<0.001*
Only CSA	63 (73.2%)	11 (32.3%)	74 (61.7%)	
CSA→Switch to others	23 (26.7%)	23 (67.6%)	46 (38.3%)	

Abbreviations: *BMI*, Body Mass Index, *HT* hypertension, *DM* diabetes mellitus, *CRD* Chronic Renal Disease, *AML* acute myeloid leukemia, *ALL* acute lymphoblastic leukemia, *MDS* myelodysplastic syndrome, *CML* chronic myeloid leukemia, *Bu-Cy* busulfan–cyclophosphamide, *Cy-TBI* cyclophosphamide–total body irradiation, *Flu-Cy-ATG* fludarabine–cyclophosphamide–anti-thymocyte globulin, *FLAMSA* fludarabine–cytarabine (Ara-C)–amsacrine, *VP-Cy-TBI* etoposide–cytarabine (Ara-C)– total body irradiation, *BEAM* BCNU (carmustine)–etoposide–cytarabine (Ara-C)–melphalan, *TBI* total body irradiation, *RT* radiotherapy, *HLA* human leukocyte antigen, *CMV* cytomegalovirus, *AHSCT* allogeneic hematopoietic stem cell transplantation. *CSA* cyclosporine

* *p* < 0.05 considered statistically significant

**Fig. 1:**
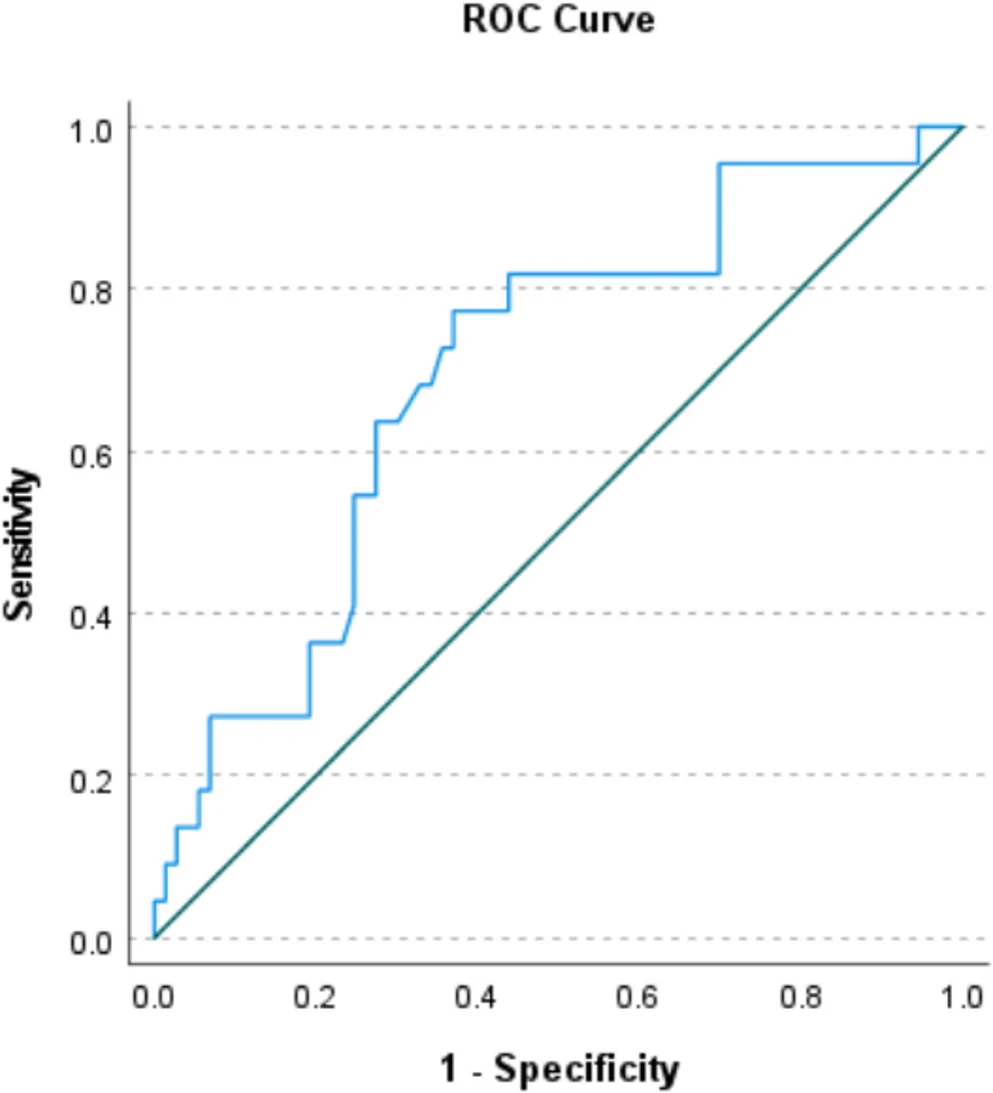
Receiver operating characteristic curve of cyclosporine levels during the first two months after allogeneic stem cell transplantation for predicting acute graft-versus-host disease. AUC, area under the curve; ROC, Receiver operating characteristic; CI, Confidince Interval; CsA, cyclosporine; aGVHD, acute graft-versus-host disease. AUC 0.697 (95% CI: 0.574–0.820, *p* = 0.005)

In the analysis evaluating the association between cyclosporine blood levels and the development of aGVHD, ROC curve analysis demonstrated a statistically significant predictive value for CsA levels during months 1–2, with an AUC of 0.697 (95% CI: 0.574–0.820, *p* = 0.005). The optimal cut-off value for months 1–2 was 274 µg/L, which corresponded to a sensitivity of 77.3% and a specificity of 63% (Fig. 1). For other time intervals, the evaluation of blood CsA levels in relation to aGVHD development using the ROC curve did not yield statistically significant results. Therefore, cut-off values for these time intervals were not specified.

Additionally, factors that may affect the development of aGVHD were evaluated using univariate and multivariate logistic regression analyses. In the univariate model, the blood CsA levels during the 1st and 2nd months (*p* = 0.008), the prophylaxis agent (only CsA or switch to others) (*p* = 0.001), pre-transplant steroid use (*p* = 0.038), and CMV seropositivity (*p* = 0.007) were identified as significant factors affecting aGVHD development (Table 2). In the reduced multivariable model, higher CsA levels during months 1–2 (OR = 0.990, 95% CI 0.981–1.000; *p* = 0.039) and pre-transplant steroid use (OR = 0.284, 95% CI 0.094–0.860; *p* = 0.026) were independently associated with lower odds of aGVHD (Table 2).

**Table 2:** Univariate and multivariate logistic regression analysis for aGVHD

Variables	Univariate Analysis OR (95% CI)	*p* value
CsA levels first 14 days	1.000 (0.993-1.007)	0.971
CsA levels first 1 month	0.997 (0.991-1.004)	0.438
CsA levels during months 1–2	0.992 (0.986-0.998)	0.008*
CsA levels during months 2-3	0.997 (0.989-1.004)	0.384
Prophylaxis Agent	1.288 (1.108-1.498)	0.001*
Pre-transplant steroid use	0.353 (0.132-0.946)	0.038*
CMV positivity	3.198 (1.383-7.396)	0.007*
Variables	**Multivariate Analysis (Full Model) OR**	***p*** ** Value**
CsA levels during months 1–2	0.991 (0.981-1.000)	0.047*
Prophylaxis Agent	1.025 (0.792-1.326)	0.851
Pre-transplant steroid use	0.293 (0.078-1.104)	0.070
CMV positivity	1.056 (0.200-5.575)	0.949
Variables	**Multivariate Analysis (Final Model) OR**	***p*** ** Value**
CsA levels during months 1–2	0.990 (0.981-1.000)	0.039*
Pre-transplant steroid use	0.284 (0.094-0.860)	0.026*

Abbreviations: *OR* odds ratio, *CI* confidence interval, *CsA* cyclosporine, *CMV* cytomegalovirus

* *p* < 0.05 considered statistically significant

## Discussion

In this single-center retrospective study, we demonstrated that CsA exposure during the 1–2 months after AHSCT plays a pivotal role in preventing aGVHD. Patients with CsA concentrations ≤ 274 µg/L during this period had a significantly higher risk of developing aGVHD, whereas CsA levels measured at other early intervals were not found to be predictive. After adjustment for potential confounders, high CsA exposure (months 1–2) and pre-transplant steroid use emerged as the only independent protective factors.

These findings highlight the importance of early, exposure-guided CsA monitoring to reduce aGVHD incidence. Although CsA has long been central to GVHD prophylaxis, no universally accepted threshold has been established due to its narrow therapeutic index, toxicity at higher concentrations, and substantial interindividual pharmacokinetic variability. Current consensus guidelines generally recommend maintaining trough concentrations between 200 and 300 ng/mL during the first post-transplant month, but reported “optimal” values differ considerably across studies, reflecting differences in conditioning regimens, donor sources, assay methods, and therapeutic drug monitoring (TDM) practices [15–17].

Our results are broadly consistent with prior reports linking inadequate CsA exposure to higher aGVHD risk, though study designs and patient populations have varied. Bianchi et al. in a large and heterogeneous adult cohort, reported that CsA levels > 195 µg/L on day 10 were associated with significantly reduced aGVHD incidence [4]. Their work provided valuable insights into very early exposure but included patients with diverse hematologic diagnoses and conditioning regimens, which may partly explain the relatively lower threshold observed.

Gupta et al. evaluated initial CsA levels within the first day’s post-transplant in acute leukemia patients and reported that early trough levels were influenced by conditioning regimen and BMI but did not predict transplant outcomes, including aGVHD [11]. Similarly, in our analysis, CsA levels measured during the first 0–14 days and the first month post-transplant were not significantly associated with aGVHD. In contrast, suboptimal CsA exposure during the 1–2 month period was strongly related to the development of aGVHD. These findings emphasize that, rather than relying solely on a single early measurement, evaluating CsA levels across different post-transplant intervals may provide a more accurate understanding of exposure dynamics and their relationship with aGVHD risk.

Moreover, Zeighami et al. using frequent twice-weekly monitoring during the first four weeks post-transplant, demonstrated that patients with lower CsA concentrations—particularly by week 3—had a significantly higher incidence of grade II–IV aGVHD [12]. Together, prior reports support the concept that inadequate CsA exposure predisposes to GVHD, though variability in patient populations and protocols contributes to heterogeneity in the timing and thresholds identified.

Taken together, the heterogeneity observed across studies [4, 11, 12] may be explained, at least in part, by differences in the kinetics of immune reconstitution after HSCT. Early immune recovery after HSCT is predominantly mediated by innate immune cells, with monocytes, granulocytes, and donor-derived NK cells recovering within the first weeks to months. In contrast, adaptive immunity—particularly CD4⁺ T-cell reconstitution—follows a much slower trajectory, often requiring 1–2 years to reach clinically significant thresholds, with some patients experiencing even more prolonged deficits [18–21]. During this vulnerable phase, insufficient CsA exposure may permit unchecked allo-reactive T-cell activity, precipitating clinically overt aGVHD [5, 6]. In line with this concept, our multivariate analysis demonstrated that higher CsA exposure during months 1–2 was independently protective against the development of aGVHD. Pre-transplant steroid use also remained independently associated with a lower risk of aGVHD, suggesting that both adequate pharmacologic immunosuppression during this critical interval and the patient’s baseline immune status contribute to shaping post-transplant alloreactivity.

Beyond these primary determinants, our univariate analyses also indicated that other factors, such as the type of prophylactic regimen (CsA alone versus switch to others) and CMV seropositivity were also associated with aGVHD incidence. However, these associations lost statistical significance in multivariate models, likely reflecting collinearity with stronger predictors or limited sample size in certain subgroups. This is consistent with prior evidence suggesting complex, bidirectional interactions between CMV status and GVHD risk. For example, a recent large retrospective analysis in patients with aplastic anemia demonstrated that higher HLA disparity and the occurrence of aGVHD were independent risk factors for early CMV reactivation, highlighting the time-dependent interplay between these variables [22]. Such findings may partly explain why CMV serostatus emerges as significant in univariate analyses but loses independent predictive value after adjustment for stronger determinants such as CsA exposure and steroid use.

### Strengths and limitations

The strengths of our study include systematic, interval-based monitoring of CsA exposure, which allowed us to capture dynamic associations rather than relying on isolated measurements. Consistent clinical follow-up ensured reliable ascertainment of aGVHD outcomes. Importantly, by restricting the cohort to patients with acute leukemia and applying the same transplant procedures uniformly to all patients, we minimized diagnostic and procedural heterogeneity, thereby enhancing the internal validity of our findings. Despite these strengths, some limitations should be acknowledged. The retrospective design, single-center setting, and relatively small sample size may limit generalizability. As an observational study, causal inferences cannot be firmly established, and center-specific practices in CsA monitoring or dose adjustment could restrict the external applicability of our results. Conditioning regimens were predominantly myeloablative, with a smaller proportion of reduced-intensity regimens; therefore, their effects were interpreted in the context of this distribution. Additionally, ROC analyses did not account for the time-dependent nature of aGVHD onset, and patients were not restricted to those at risk within each time window, which may have introduced bias in estimating predictive performance.

## Conclusion

In conclusion, our study demonstrates that maintaining CsA concentrations above 274 µg/L during the 1–2 months following AHSCT is critical for reducing the incidence of aGVHD. While several variables were associated with aGVHD risk in univariate analyses, only higher CsA exposure during this critical interval and pre-transplant steroid use remained independent protective factors in multivariate models. These findings underscore the importance of standardized, interval-based therapeutic drug monitoring and tailored immunosuppressive strategies to optimize post-transplant outcomes in hematology practice.

## Data Availability

The data that support the findings of this study are not openly available due to reasons of sensitivity and are available from the corresponding author upon reasonable request. The data were obtained from patient records at Bursa Uludag University Faculty of Medicine Hospital, Hematopoietic Stem Cell Transplantation Unit.
